# Intracellular S100A9 Promotes Myeloid-Derived Suppressor Cells during Late Sepsis

**DOI:** 10.3389/fimmu.2017.01565

**Published:** 2017-11-17

**Authors:** Jun Dai, Ajinkya Kumbhare, Dima Youssef, Charles E. McCall, Mohamed El Gazzar

**Affiliations:** ^1^Department of Internal Medicine, East Tennessee State University College of Medicine, Johnson City, TN, United States; ^2^Section of Molecular Medicine, Department of Internal Medicine, Wake Forest University School of Medicine, Winston-Salem, NC, United States

**Keywords:** sepsis, myeloid-derived suppressor cells, immune suppression, S100A9, myeloid cell reprogramming

## Abstract

Myeloid precursor cell reprogramming into a myeloid-derived suppressor cell (MDSC) contributes to high mortality rates in mouse and human sepsis. S100A9 mRNA and intracellular protein levels increase during early sepsis and remain elevated in Gr1^+^CD11b^+^ MDSCs after pro-inflammatory sepsis transitions to the later chronic anti-inflammatory and immunosuppressive phenotype. The purpose of this study was to determine whether intracellular S100A9 protein might sustain Gr1^+^CD11b^+^ MDSC repressor cell reprogramming during sepsis. We used a chronic model of sepsis in mice to show that S100A9 release from MDSCs and circulating phagocytes decreases after early sepsis and that targeting the *S100a9* gene improves survival. Surprisingly, we find that intracellular S100A9 protein translocates from the cytosol to nucleus in Gr1^+^CD11b^+^ MDSCs during late sepsis and promotes expression of miR-21 and miR-181b immune repressor mediators. We further provide support of this immunosuppression pathway in human sepsis. This study may inform a new therapeutic target for improving sepsis outcome.

## Introduction

Sepsis is the leading cause of death among critically ill patients (1, 2). The initial/acute phase of sepsis, which may cause cardiovascular collapse and rapid death, more commonly progresses to chronic sepsis, which is clinically characterized by innate and adaptive immune incompetence and organ failure (3–5). This chronic sepsis state has been named the persistent inflammation-immunosuppression and catabolism syndrome (PICS) (6). Although well recognized and characterized, the molecular events that promote this unresolving state of sepsis are unclear, but sustained presence of a cell phenotype called myeloid-derived suppressor cells (MDSCs) likely contributes to PICS (7). This broad-based immune-suppressor state with organ failure is typified by repressed effector phenotypes of CD4^+^ and CD8^+^ T-cells, circulating, splenic, and myeloid- dendritic, NK cells and other heterogeneous innate and adaptive immune cells (4, 8). PICS clinical phenotype is reflected by persistent bacterial infection and reactivation of latent viruses (4, 6). Importantly, the immune-suppressor phenotype typifies endotoxin tolerance and develops within hours of the early hyper-inflammatory cytokine-mediated “cytokine storm” (9, 10). Other characteristics are increased apoptosis of different cell types (4) and repressed mitochondrial fueling by glucose and fatty acids (11, 12).

Calcium-binding proteins S100A8 and S100A9 are markedly increased during sepsis (13), and their elevated levels in circulating innate immune phagocytes during sepsis may contribute to acute and chronic inflammation (14, 15). S100A8 and S100A9 are mainly expressed in the myeloid lineage cells (16), including monocytes and granulocytes, but not resident tissue macrophages (15) and are dominant in the systemic circulation. The two proteins are also expressed in early stages of myeloid differentiation and decline during maturation, but remain elevated in neutrophils (17). S100A8/A9 protein complexes are mostly cytosolic, where they are recruited to the plasma membrane and secreted after protein kinase C (PKC) activation and cytoskeletal rearrangement following phagocytosis (14).

Most studies of S100A8/A9 proteins emphasize them as pro-inflammatory mediators, which amplify acute and chronic inflammatory processes during phagocyte activation (14, 18, 19). S100A8/A9 heterodimers released at sites of inflammation induce more S100A8/A9 in phagocytes, thereby acting as a feedforward loop to amplify the local inflammatory reaction (15, 20). Heterodimeric S100A8/A9 binds to and activates toll-like receptor 4 (TLR4), a master “alarmin” sensor (21). S100A8/A9 heterodimers are secreted readily from bone marrow cells and blood monocytes following bacterial lipopolysaccharide (LPS)-mediated activation of TLR4 (14). Further supporting pro-inflammatory functions is the finding that S100A9^−/−^ mice exhibit blunted LPS-induced endotoxemia (21). In contrast, there are reports of anti-inflammatory properties of S100A8/A9 showing that intraperitoneal injections of S100A8/A9 heterodimers into rat after LPS administration reduces serum levels of pro-inflammatory IL-6 and nitrite (22). In addition, because S100A8/A9 supports phagocyte migration, inhibiting S100A8/A9 would have anti-inflammatory potential (14). An important finding suggesting a repressor function is that immune repressor mediator IL-10 induces S100A8 in human macrophages (23).

Here, we report a new function for S100A9 that further supports the repressor concept of S100A8/A9. We find that mice genetically deficient for S100A9 are protected from late sepsis deaths by preventing MDSC repressor activity. We further find that S100A8/A9 proteins are released from phagocytes during early, but not late sepsis, and that cytosolic S100A9 translocates from cytosol to the nucleus of MDSCs. Mechanistically, our data support that nuclear S100A9 promotes the expression of known immunosuppressive miR-21 and miR-181b (24). The findings of this study support therapeutic targeting of S100A9 for chronic sepsis.

## Materials and Methods

### Mice

The S100a9 mutant mouse strain [S100a9tm1^(KOMP)/VLcg^] used for this study was created from ES cell clone 12158a-E1, generated by Regeneron Pharmaceuticals (Eastview, NY, USA), and made into live mice by the KOMP Repository^1^ and the Mouse Biology Program^2^ at the University of California Davis. Methods used to create the Velocigene targeted alleles have been published (25). Cryopreserved sperm from the KOMP repository was used for *in vitro* fertilization of C57BL/6NJ oocytes to reconstitute the strain (work performed by TransViragen, Chapel Hill, NC, USA). Heterozygous animals were intercrossed to generate homozygous (−/−) mutant animals for study. The mice were bred and housed in a pathogen-free facility in the Division of Laboratory Animal Resources. Wild-type male C57BL/6J mice, 8–10 weeks were purchased from Jackson Laboratory (Bar Harbor, ME, USA) and used as controls, and were acclimated to the new environment for a week before surgery. All experiments were conducted in accordance with National Institutes of Health guidelines and were approved by the East Tennessee State University Animal Care and Use Committee.

### Polymicrobial Sepsis

Polymicrobial sepsis was induced in male wild-type and S100A9 knockout mice, 8–10 week old, by cecal ligation and puncture (CLP) as described previously (26). Briefly, mice were anesthetized *via* inhalation with 2.5% isoflurane (Abbott Laboratories, Abbott Park, IL, USA). A midline abdominal incision was made and the cecum was exteriorized, ligated distal to the ileocecal valve, and then punctured twice with a 23-gauge needle. A small amount of feces was extruded into the abdominal cavity. The abdominal wall and skin were sutured in layers with 3-0 silk. Sham-operated mice were treated identically except that the cecum was neither ligated nor punctured. Mice received (i.p.) 1 ml lactated Ringers plus 5% dextrose for fluid resuscitation. To induce sepsis that develops into early and late phases, mice were subcutaneously administered antibiotic (Imipenem; 25 mg/kg body weight) or an equivalent volume of 0.9% saline. To establish intra-abdominal infection and approximate the clinical condition of early human sepsis where there is a delay between the onset of sepsis and the delivery of therapy (27), injections of Imipenem were given at 8 and 16 h after CLP. Based on our experience, these levels of injury and manipulation create prolonged infections with high mortality (~60–70%) during the late/chronic phase (26).

The presence of early sepsis was confirmed by transient systemic bacteremia and elevated cytokine levels in the first 5 days after CLP. Late/chronic sepsis (after day 5) was confirmed by enhanced peritoneal bacterial overgrowth and reduced circulating pro-inflammatory cytokines. Table S1 in Supplementary Material includes the CLP mice that were used in the study.

### Sepsis Patients

Patients 18 years of age or older who were admitted to Johnson City Medical Center and Franklin Woods Hospital in Johnson City, Tennessee, and who were diagnosed with sepsis or septic shock were included in the study. Sepsis was defined as the presence of suspected or documented infection with at least two of the following criteria: core temperature >38°C or <36°C; heart rate >90 beats/min; respiratory rate >20 breaths/min or arterial blood partial pressure of carbon dioxide <32 mmHg; or white blood cell count >12,000 cells/mm^3^ or <4,000/mm^3^. Septic shock was defined as sepsis with persisting hypotension requiring vasopressors to maintain MAP ≥65 mmHg and having a serum lactate >2 mmol/L despite adequate volume resuscitation (28). Patients presented with infections related to Gram-negative or Gram-positive bacteria. The primary infection included urinary tract infection, blood stream infection, and respiratory tract infection. Patients had at least 1 comorbid condition, including nephropathy, psoriasis, splenectomy, colon cancer, or pulmonary aspergillosis. Patients with leukopenia due to chemotherapy or glucocorticoid therapy or HIV infection were excluded from the study. Patients were divided into two categories: early sepsis and late sepsis, relative to the day of sepsis diagnosis. The early septic group included patients within 1–5 days of sepsis diagnosis. Those who have been septic for more than 6 days were considered late septic. For this latter group, blood was drawn at days 6–68 after sepsis diagnosis. Blood samples obtained from healthy control subjects were supplied by Physicians Plasma Alliance (Gray, TN, USA). The study was approved by the Institutional Review Board (IRB) of the East Tennessee State University (IRB#: 0714.6s). Signed informed consent was obtained from all subjects.

### Immunoblotting

Whole cell lysate, cytoplasmic, and nuclear proteins were prepared as described previously (29). Equal amounts of protein extracts were mixed with 5× Laemmeli sample buffer, separated by SDS-10% polyacrylamide gel (Bio-Rad, Hercules, CA, USA) and subsequently transferred to nitrocellulose membranes (Thermo Fisher Scientific, Waltham, MA, USA). After blocking with 5% milk in Tris-buffered saline/Tween-20 for 1 h at room temperature, membranes were probed overnight at 4°C with the following primary antibodies: anti-goat S100A8 (sc-8112; Santa Cruz Biotechnology, Santa Cruz, CA, USA); anti-Rabbit S100A9 (ab75478) and anti-mouse S100A8/A9 heterodimer (ab17050), both from Abcam (Cambridge, MA, USA); anti-Rabbit phospho-threonine113 S100A9 (12782; Signalway Antibody LLC., College Park, MD, USA); anti-Rabbit p38 (sc-7149) and p-p38 (sc-17852-R), anti-mouse phospho-serine 657 PKC (sc-377565), anti-Rabbit phospho-serine 660 PKC (sc-11760-R) (Santa Cruz Biotechnology). The S100A8/9 heterodimer was detected under non-denaturing conditions. After washing, blots were incubated with the appropriate HRP-conjugated secondary antibody (Life Technologies, Grand Island, NY) for 2 h at room temperature. Proteins were detected with the enhanced chemiluminescence detection system (Thermo Fisher Scientific, Waltham, MA, USA), the bands were visualized using the ChemiDoc XRS System (Bio-Rad), and the images were captured with the Image Lab Software V3.0. Membranes were stripped and re-probed with actin (Sigma-Aldrich, Saint Louis, MO, USA) antibody as a loading control.

### Blood Phagocytes and Plasma

Mice were subjected to deep anesthesia with isoflurane and blood was collected *via* cardiac puncture using EDTA-rinsed syringes. Blood was diluted fivefold (up to 2.5 ml) in phosphate-buffered saline containing 0.3 mM EDTA. To obtain phagocytes (mainly monocytes and polymorphonuclear neutrophils), we first isolated peripheral blood mononuclear cells (PBMCs) and granulocytes by gradients of Histopage-1077 and Histopaque-1119 following the manufacturer’s instructions (Sigma-Aldrich, Saint Louis, MO, USA). Briefly, diluted blood (2.5 ml) was layered in a 15-ml conical tube containing 2.5 ml Histopague-1119 (bottom layer) and 2.5 ml Histopaque-1077. Samples were centrifuged at 700*g* for 30 min at room temperature. Plasma was collected and stored at −20°C for later analysis. The upper cell layer containing PBMCs was removed and washed with PBS. The lower cell layer containing granulocytes was washed with PBS three times by centrifugation at 200*g* for 10 min. With this method, erythrocytes and platelets are removed by the low speed centrifugation during the washing steps. Monocytes were then isolated from the PBMCs by positive selection with anti-Ly6C antibody and combined with the granulocytes (mainly neutrophils), to represent the phagocyte population used in the study. In some experiments, granulocytes were used separately.

### Peritoneal Bacterial Quantification

Immediately after mice were euthanized, the peritoneal cavity was lavaged with 5 ml PBS. The lavage was cleared by centrifugation and plated on trypticase soy agar base (BD Biosciences, Sparks, MD, USA). The plates were incubated for 24 h at 37°C under aerobic conditions. The plates were read by a microbiologist and the colony-forming units (CFUs) were enumerated.

### Isolation of Gr1^+^CD11b^+^ Cells

Gr1^+^CD11b^+^ cells were isolated from the bone marrow or spleens by use of magnetically assisted cell sorting according to the manufacturer’s protocol (Miltenyi Biotech, Auburn, CA, USA). The bone marrow was flushed out of the femurs with RPMI-1640 medium (without serum) under aseptic conditions (26). The spleens were dissociated in RPMI-1640 medium. A single cell suspension of the bone marrow or spleens was made by pipetting up and down and filtering through a 70-µm nylon strainer, followed by incubation with erythrocyte lysis buffer and washing. To purify total Gr1^+^CD11b^+^ cells, the single cell suspension was subjected to positive selection of the Gr1^+^ cells by incubating with biotin-coupled mouse anti-Gr1 antibody (Clone RB6-8C5; eBioscience, San Diego, CA, USA) for 15 min at 4°C. Cells were then incubated with anti-biotin magnetic beads for 20 min at 4°C and subsequently passed over a MS column. Purified Gr1^+^CD11b^+^ cells were then washed and resuspended in sterile saline. The cell purity was more than 90% as determined by flow cytometry.

### Flow Cytometry

Gr1^+^CD11b^+^ cells were stained by incubation for 30 min on ice in staining buffer (PBS plus 2% FBS) with the following mouse antibodies: fluorescein isothiocyanate (FITC)-conjugated anti-Gr1, phycoerythrin (PE)-conjugated anti-CD11b, allophycocyanin-conjugated anti-F4/80, PE-conjugated anti-CD11c, FITC-conjugated anti-MHC II. CD4^+^ T cells were stained with PE-conjugated anti-CD4 antibody (all antibodies were from eBioscience, San Diego, CA, USA). An appropriate isotype-matched control was used for each antibody. After washing, the samples were analyzed by an Accuri C6 flow cytometer (BD, Franklin Lakes, NJ, USA).

### Cell Proliferation Assay

Spleen CD4^+^ T cells were isolated from normal (naive) mice by positive selection using magnetic beads (Miltenyi). Cells were fluorescently labeled with carboxy-fluorosceindiacetate, succinimidyl ester (CFSE) dye using the Vybrant CFDA SE Cell Tracer Kit (Invitrogen Molecular Probes, Eugene, OR, USA). Cells were incubated for 10 min at room temperature with 10 µM CFSE dye and then cocultured (at 1:1 ratio) with the Gr1^+^ CD11b^+^ cells, which were isolated from the bone marrow of sham or late septic mice. T cell proliferation was induced by the stimulation with an anti-CD3 plus anti-CD28 antibody (1 μg/ml/each). After 3 days, cells were harvested and CD4^+^ T cell proliferation was determined by the step-wise dilution of CFSE dye in dividing CD4^+^ T cells, using flow cytometry.

### Real-time PCR

Quantitative real-time qPCR was used to determine the expression levels of S100A8, S100A9, miR-21, and miR-181b in Gr1^+^CD11b^+^ cells. For S100A8 and S100A9, total RNA was isolated using TRIzol reagent (Invitrogen, Carlsbad, CA, USA) and amplified using QuantiNova SYBR Green RT-PCR kit and QuantiTect Primer Assays specific to S100A8 and S100A9 (Qiagen, Germantown, MD). The target RNA: 18S rRNA ratio was calculated using the 2^−ΔΔCt^ cycle threshold.

For miR-21 and miR-181b measurements, miRNA-enriched RNA was isolated and measured using miScript SYBR Green PCR kit and miScript Primer Assays specific to miR-21 and miR-181b (Qiagen). The expression level was calculated using the 2^−ΔΔCt^ cycle threshold method after normalization to the endogenous U6 RNA as an internal control.

### Ca^2+^ Assay

Intracellular calcium levels in phagocytes were measured using the cell-based Fluo-4 NW Calcium Assay kit according to the manufacturer’s protocol (Molecular Probes, Eugene, OR, USA).

### ELISA

Specific ELISA kits were used to measure the levels of S100A8, S100A9 (MyBioSource, San Diego, CA, USA) and S100A8/A9 heterodimer (R&D Systems, Minneapolis, MN, USA). Levels of TNFα and IL-10 were determined using specific ELISA kits (eBioscience) according to the manufacturer’s instructions. Each sample was run in duplicate.

### Statistical Analysis

The Kaplan–Meier survival curve was plotted by use of a GraphPad Prism 5 (GraphPad Software, La Jolla, CA, USA) and survival significance was determined by a log-rank test. Other data were analyzed by Microsoft Excel, V3.0. Data are expressed as mean ± SD. Differences among groups were analyzed by a two-tailed Student’s *t*-test for two groups and by ANOVA for multiple groups. *P* values < 0.05 were considered statistically significant.

## Results

### S100A8/9 Play a Major Role in Sepsis Mortality

**Figure 1:**
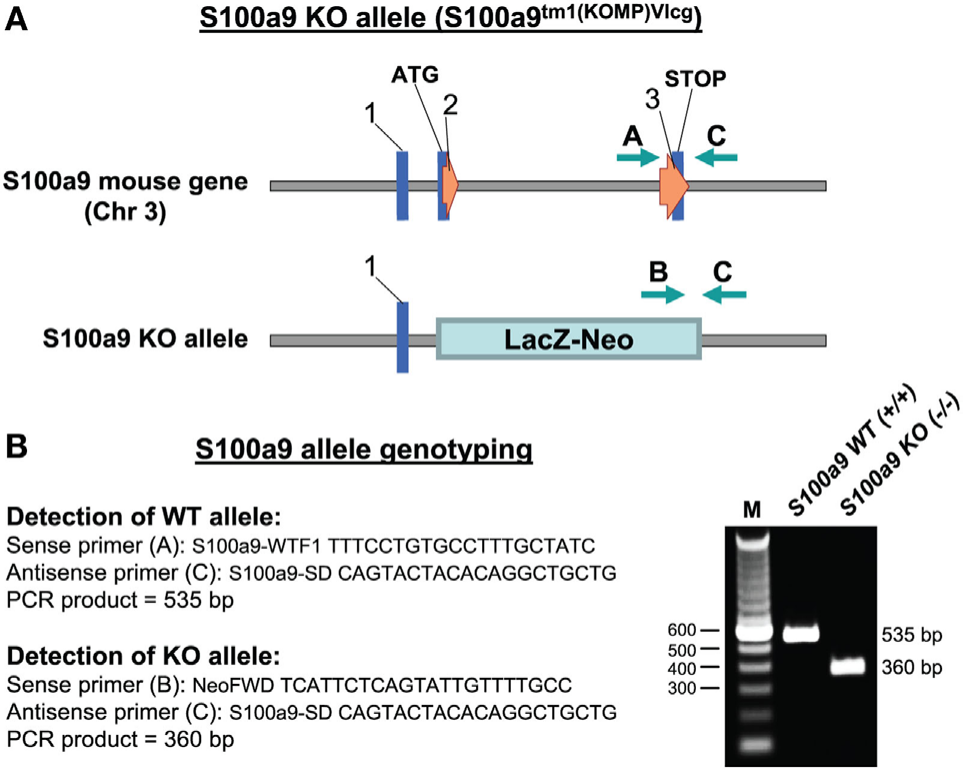
Targeted deletion of the *S100a9* gene. **(A)** The *S100a9* gene was inactivated by insertion of the lacZ-neo cassette into exon 2 immediately next to the transcription start site (ATG). **(B)** Nucleotide sequences of the genotyping primers used for detection of the WT and KO alleles and agarose gel electrophoresis of the PCR products generated from Gr1^+^CD11b^+^ cells (right). M, size marker; WT, wild-type; KO, knockout.

To examine the physiologic contribution of S100A8/A9 to sepsis, we created an S100A9 knockout mouse on a C57BL/6 background. A diagram showing the *S100a9* knockout allele and location of the PCR genotyping primers is presented in Figure 1.

Our model of polymicrobial sepsis with fluid resuscitation and limited antibiotic treatment develops into early and late sepsis phases (26). We previously showed that mortality is higher during the late/chronic phase and is mainly due to immunosuppression (30). Sepsis was induced by CLP, and survival was reported for a 4-week period. Mice moribund during early sepsis (defined as the first 5 days after CLP) or late sepsis (the time after day 6) were sacrificed and analyzed. Because most of the S100A9 knockout mice survived late sepsis, for each moribund and sacrificed mouse from the wild-type group, a healthy appearing mouse from the S100A9 knockout group was also sacrificed at the same time, but is reported as “survivor.”

**Figure 2:**
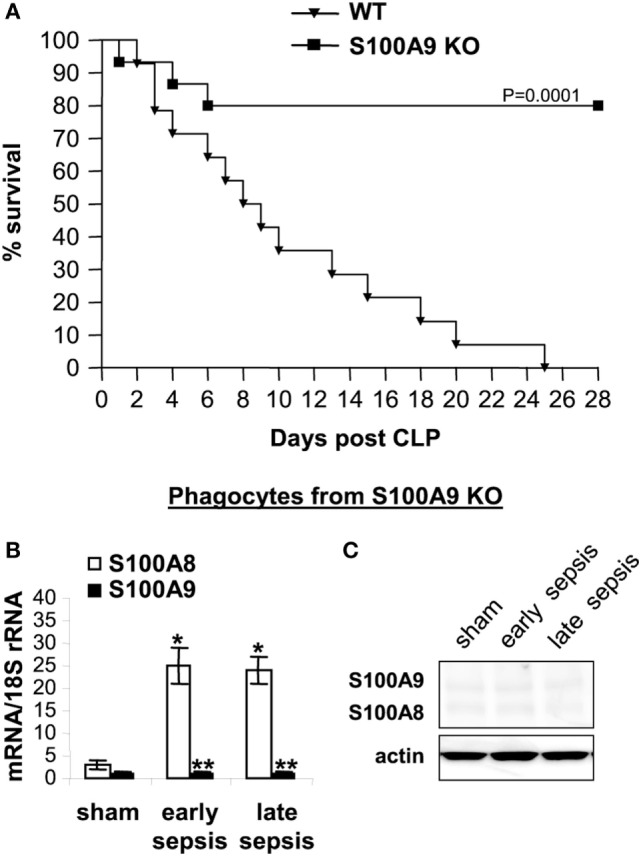
The S100A9 knockout improves late sepsis survival. Sepsis was induced by cecal ligation and puncture (CLP) using a 23-gauge needle, and mice were given antibiotics with fluid resuscitation. With this injury and treatment, sepsis develops into an early phase (defined as days 1–5 after CLP) and a late phase (day 6 thereafter). **(A)** Kaplan–Meier survival curve (*n* = 22 mice per group). Mortality was monitored for 28 days and the deaths were separated into two categories: early deaths (those occurring on days 1–5) and late deaths (those occurring on days 6–28). All moribund mice suffered significant weight loss (>30%), hypothermia (<34°C) and loss of righting reflex. Each group included 30 mice. Mice that died spontaneously (i.e., did not meet the criteria for morbundity) were excluded. The results are representative of three experiments. Mice used in all subsequent experiments were pooled from three experiments. **(B)** S100A8 and S100A9 mRNA levels. Blood was collected from moribund mice that were killed at days 1–5 (representing early sepsis) or days 6–25 post CLP (representing late sepsis). During late sepsis, a corresponding number of surviving, healthy appearing S100A9 KO mice were also sacrificed and analyzed at the same time, but are reported as “survivors.” Phagocytes were isolated from peripheral blood by gradients of Histopage-1077 and Histopaque-1119. Total RNA was extracted and analyzed by real-time PCR. The S100A8 and S100A9 expression levels were normalized to 18S rRNA. Data are means ± SD (*n* = 6–8 mice per group), and are representative of three experiments (*p < 0.001 vs. sham; **p < 0.001 vs. S100A8). **(C)** Levels of S100A8 and S100A9 proteins in phagocyte lysate were determined by immunoblotting. The membranes were first incubated with anti-S100A8 antibody, followed by anti-S100A9 antibody. Cells were pooled from 2–3 mice per group, and the results are representative of two immunoblots. WT, wild-type; KO, knockout.

As shown in Figure 2A, 100% of the wild-type mice died before the end of the 4-week period. Lack of S100A8/A9 proteins slightly improved survival during early sepsis at days 3–5, with 14.7 and 15.2% increases in survival, respectively, at days 3 and 5 compared to the wild-type mice. Of note, survival in the late sepsis phase was improved by 80% in the S100A9 knockout (S100A9^−/−^) vs. wild-type mice (Figure 2A). As expected, S100A8 mRNA, but not protein, was detected in phagocytes from septic wild-type and S100A9 knockout mice (Figures 2B,C).

### S100A9 Knockout Attenuates Late Sepsis Immunosuppression

**Figure 3:**
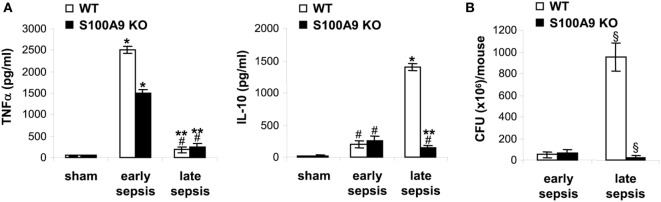
The S100A9 knockout mice do not develop immunosuppressive phenotypes during late sepsis. **(A)** Decreased production of the immunosuppressive cytokine IL-10 in S100A9 knockout mice during late sepsis. Blood was collected from moribund mice undergoing early and late sepsis. The early and late sepsis groups, respectively, included mice that were killed between days 1–5 and 6–28 after cecal ligation and puncture. During late sepsis, a corresponding number of surviving, healthy appearing S100A9 KO mice were also killed and analyzed at the same time. Plasma was collected and levels of TNFα and IL-10 were measured by ELISA. **(B)** Diminished peritoneal bacterial growth in the S100A9 knockout mice during late sepsis. Mice were subjected to peritoneal lavaging immediately after sacrifice. Lavage bacteria were cultured on tryptase soy agar plates and the colony-forming units (CFUs) were enumerated 24 h later. Data are means ± SD (*n* = 5–7 mice per group), and are representative of three experiments. *p < 0.0001 vs. sham; ^#^*p* < 0.05 vs. sham; **p < 0.0002 vs. early sepsis (TNFα) or late sepsis WT (IL-10); ^§^*p* < 0.001 vs. early sepsis or late sepsis WT **(B)**. WT, wild type; KO, knockout.

Early/acute septic mice display elevated levels of pro-inflammatory cytokines such as TNFα, whereas late/chronic septic mice are known to progress to an anti-inflammatory/immunosuppressive phenotype characterized by elevated levels of IL-10 and increased peritoneal bacterial growth (30). We measured levels of IL-10 and the pro-inflammatory cytokine TNFα in the plasma, as well as levels of peritoneal bacteria. As shown in Figure 3A, TNFα levels increased in both wild-type and S100A9 knockout mice during early sepsis, but were significantly higher in wild-type mice. During late sepsis, however, TNFα significantly decreased in both wild-type and knockout mice. In contrast, wild-type and S100A9 knockout mice produced small amounts of the immunosuppressive IL-10 during early sepsis, but significantly increased during late sepsis in the wild-type mice and not in knockout mice (Figure 3A). Peritoneal bacteria significantly increased in wild-type mice during late sepsis, but diminished in the S100A9 knockout mice (Figure 3B). These results support that improved sepsis survival in the S100A9 knockout mice parallels reduced immunosuppressive cytokine production and increased local bacterial clearance. These findings suggest that S100A9 expression may promote late sepsis immunosuppression and predicts that its genetic reduction would limit MDSC repressor cells.

### S100A9 Deficiency Prevents MDSC Development during Sepsis

**Figure 4:**
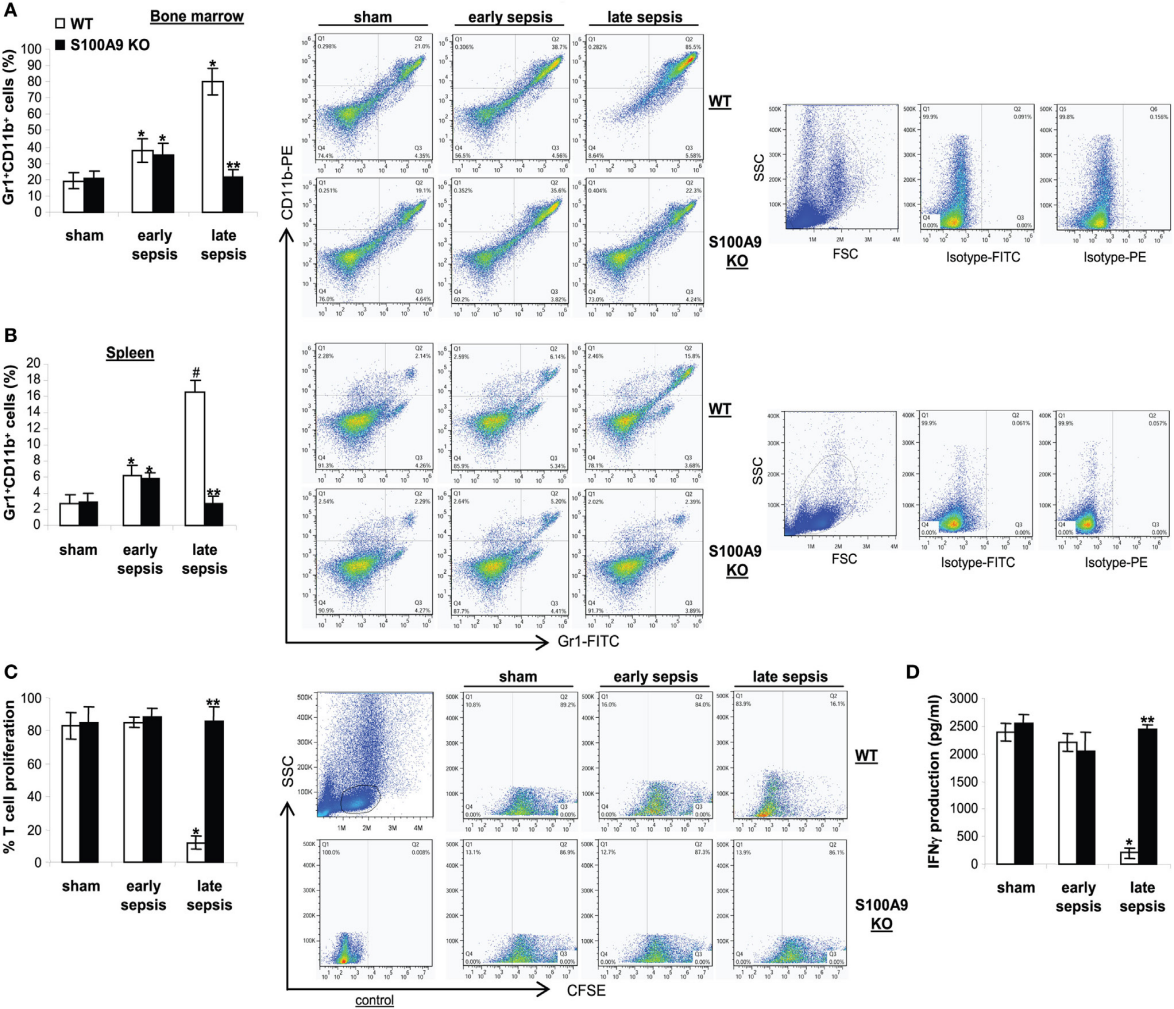
The S100A9 knockout mice do not generate immunosuppressive Gr1^+^CD11b^+^ myeloid-derived suppressor cells (MDSCs). Sepsis was induced by cecal ligation and puncture (CLP), and mice were treated and sampled as described in Figure 2. The early and late sepsis groups, respectively, included mice that were killed between days 1–5 and 6–28 after CLP. **(A,B)** Inhibition of MDSC expansion in the S100A9 knockout mice during late sepsis. Bone marrow **(A)** and spleen cells **(B)** cells were harvested, stained with anti-Gr1 and anti-CD11b antibodies and analyzed by flow cytometry. Representative dot plots (right) and percentages of cells gated on Gr1 and CD11b staining are shown. *p < 0.05 vs. sham; ^#^*p* < 0.001 vs. sham; **p < 0.001 vs. WT. **(C)** Effect of MDSCs on T cell proliferation. To determine the immunosuppressive effect of sepsis Gr1^+^ CD11b^+^ MDSCs, spleen CD4^+^ T cells were isolated from normal (naive) mice and labeled with the fluorescent dye carboxy-fluorosceindiacetate, succinimidyl ester (CFSE) for 10 min at room temperature. Gr1^+^ CD11b^+^ cells were then cocultured (at 1:1 ratio) with CD4^+^ T cells. The culture was stimulated with anti-CD3 plus anti-CD28 antibodies (1 μg/ml/each). After 3 days, CD4^+^ T cell proliferation was determined by the step-wise dilution of CFSE dye in dividing CD4^+^ T cells by flow cytometry. Percentages of cell proliferation were calculated as follow: % cell proliferation = 100 × (count from T cell + Gr1^+^CD11b^+^ cell culture/count from T cell culture). *p < 0.001 vs. sham or early sepsis; **p < 0.001 vs. late sepsis WT. **(D)** The culture supernatants were analyzed for IFNγ levels by ELISA. *p < 0.001 vs. sham or early sepsis; **p < 0.0001 vs. late sepsis WT. Data are means ± SD (*n* = 4–6 mice per group) and are representative of three experiments. WT, wild type; KO, knockout.

Because S100A9 knockout prevented late sepsis deaths, we first tested whether S100A9 deficiency affects MDSC generation during sepsis response. We determined MDSC numbers in sham and septic mice. Numbers of Gr1^+^CD11b^+^ cells significantly increased in the bone marrow in wild-type and S100A9 knockout mice in early sepsis (Figure 4A), as we reported previously (30), but were significantly decreased in the S100A9 knockout mice in late sepsis (Figure 4A). We observed similar patterns of Gr1^+^CD11b^+^ cell expansion in the spleens, but the numbers were far less compared with the bone marrow (Figure 4B).

It is known that Gr1^+^CD11b^+^ MDSCs from late, but not early, septic mice suppress T cell activation and proliferation (31). Accordingly, we investigated effects of Gr1^+^CD11b^+^ cells on T cell proliferation and activation in wild-type and S100A9 knockout mice, as assessed by IFNγ production. Spleen CD4^+^ T cells from naive, wild-type mice were cocultured with Gr1^+^CD11b^+^ cells from sham (as a control) and septic mice, and then stimulated with anti-CD3 and anti-CD28 antibodies in order to activate the antigen receptor and assess immune competence. Gr1^+^CD11b^+^ cells from early septic wild-type or S100A9 knockout mice could not suppress CD4^+^ T cell proliferation but exhibited slightly reduced IFNγ production (Figures 4C,D). In contrast, Gr1^+^CD11b^+^ cells from late septic wild-type mice significantly decreased CD4^+^ T cell proliferation and IFNγ production compared with Gr1^+^CD11b^+^ cells from sham or early septic mice. Moreover, Gr1^+^CD11b^+^ cells from late septic knockout mice could not inhibit T cell proliferation, nor could they reduce IFNγ production after antigen stimulation by anti-CD3 and anti-CD28 (Figures 4C,D). These results suggest that S100A9 promotes MDSC repressor function during chronic sepsis. Next, we investigated the S100A8/9 biology in septic mice.

### S100A8/A9 Secretion from Phagocytes Decreases in Late Septic Mice

**Figure 5:**
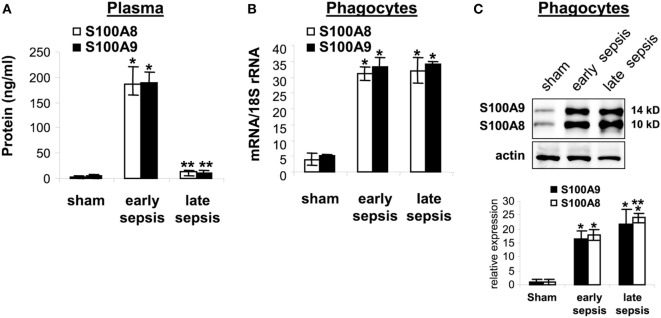
The S100A8 and S100A9 protein secretion is inhibited during late sepsis despite normal expression levels. **(A)** Secreted S100A8 and S100A9 proteins was determined by measuring their plasma levels using ELISA. **(B)** Levels of S100A8 and S100A9 mRNAs. Phagocytes were isolated from peripheral blood by gradients of Histopage-1077 and Histopaque-1119, and mRNA levels were determined by real-time PCR. The S100A8 and S100A9 expression levels were normalized to 18S rRNA. Data are means ± SD (*n* = 5–9 mice per group), and are representative of three experiments (*p < 0.001 vs. sham; **p < 0.001 vs. early sepsis). **(C)** Levels of S100A8 and S100A9 proteins in phagocyte lysate were determined by immunoblotting. The membranes were first incubated with anti-S100A8 antibody. After washing (without stripping), the membranes were incubated with anti-S100A9 antibody. Cells were pooled from 2–3 mice per group. The early and late sepsis groups, respectively, included mice that were killed between days 1–5 and 6–28 after cecal ligation and puncture. Representative blot (upper) and densitometric analysis of blots from three experiments (lower) are shown. Values were normalized to β-actin, and are presented relative to sham (*p < 0.001 vs. sham; **p < 0.05 vs. early sepsis).

Having linked gene expression to sepsis outcome, we more closely assessed these proteins during sepsis. First, we tested whether S100A8/A9 proteins accumulate in plasma obtained from sham and septic mice during early and late sepsis. Both S100A8/A9 protein levels increased in plasma during early sepsis, but decreased during late sepsis (Figure 5A). Similar patterns of S100A8/A9 heterodimer secretion occurred (data not shown). We then determined whether S100A8 and S100A9 gene expressions paralleled plasma levels during early and late sepsis by measuring mRNA and protein in circulating phagocytes (mainly monocytes and neutrophils), the primary source of S100A8/A9 (14). S100A8/A9 mRNA and protein levels, in contrast to plasma levels, were similar in circulating phagocytes during early and late septic mice (Figures 5B,C). These results support that S100A8/A9 expression increases in mature phagocytes during early and late sepsis, while protein secretion decreases or extracellular clearance increases.

### S100A9 Secretion from Gr1^+^CD11b^+^ MDSC Decreases during Late Sepsis

**Figure 6:**
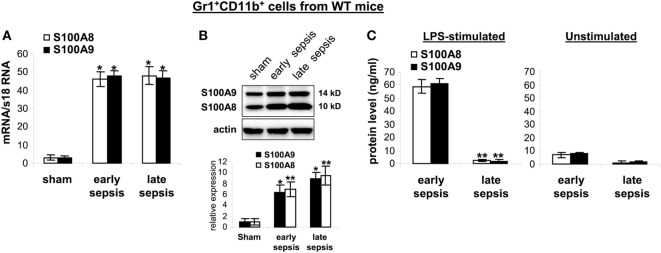
The S100A8 and S100A9 proteins are retained in Gr1^+^CD11b^+^ cells during late sepsis. Gr1^+^CD11b^+^ cells were isolated from the bone marrow cells by positive selection. The early and late sepsis groups, respectively, included mice that were killed between days 1–5 and 6–28 after cecal ligation and puncture. **(A)** Levels of S100A8 and S100A9 mRNAs. Total RNA was extracted from Gr1^+^CD11b^+^ cells, and mRNA levels were determined by real-time PCR. The S100A8 and S100A9 expression levels were normalized to 18S rRNA (*p < 0.001 vs. sham). **(B)** Levels of S100A8 and S100A9 proteins in Gr1^+^CD11b^+^ whole cell lysates were determined by immunoblotting. Cells were pooled from 2–3 mice per group. Representative blot (upper) and densitometric analysis of blots from three experiments (lower) are shown. Values were normalized to β-actin and are presented relative to sham (*p < 0.01 vs. sham; **p < 0.05 vs. sham). **(C)** Protein secretion after stimulation with lipopolysaccharide (LPS). Gr1^+^CD11b^+^ cells were isolated from the bone marrow of septic mice and stimulated for 24 h with 1 µg/ml LPS (*E. coli* serotype 0111:B4). Levels of S100A8 and S100A9 in the culture supernatants were determined by ELISA. Data in *A* and *C* are means ± SD (*n* = 5–6 mice per group), and are representative of three experiments (**p < 0.0003 vs. early sepsis).

The results presented above suggested that secreted or intracellular S100A8/A9 protein may be needed to generate the MDSC repressor phenotype. To examine this question, we first measured mRNA and protein levels in Gr1^+^CD11b^+^ cells isolated from the bone marrow of wild-type mice with or without sepsis. S100A8/A9 mRNAs similarly increased during early and late sepsis (Figure 6A). Immunoblotting revealed that S100A8/A9 protein levels also increased during early sepsis and accumulated in late sepsis Gr1^+^CD11b^+^ cells (Figure 6B). We next studied release of S100A8/A9 from MDSCs.

Stimulation of bone marrow myeloid cells with bacterial endotoxin LPS induces release of S100A8/A9 proteins from the cytosol (21). We found that Gr1^+^CD11b^+^ cells from early septic mice released large amounts of S100A8/A9 proteins after stimulation with LPS (Figure 6C). In contrast, S100A8/A9 protein release from late sepsis Gr1^+^CD11b^+^ MDSCs diminished after LPS stimulation. This unexpected finding suggests a dichotomous regulation of S100A8/A9 release with intracellular accumulation during late vs. early sepsis.

### S100A9 Protein Phosphorylation and Dimerization Is Inhibited, and the Protein Is Translocated to the Nucleus in Late Sepsis Gr1^+^CD11b^+^ MDSCs

**Figure 7:**
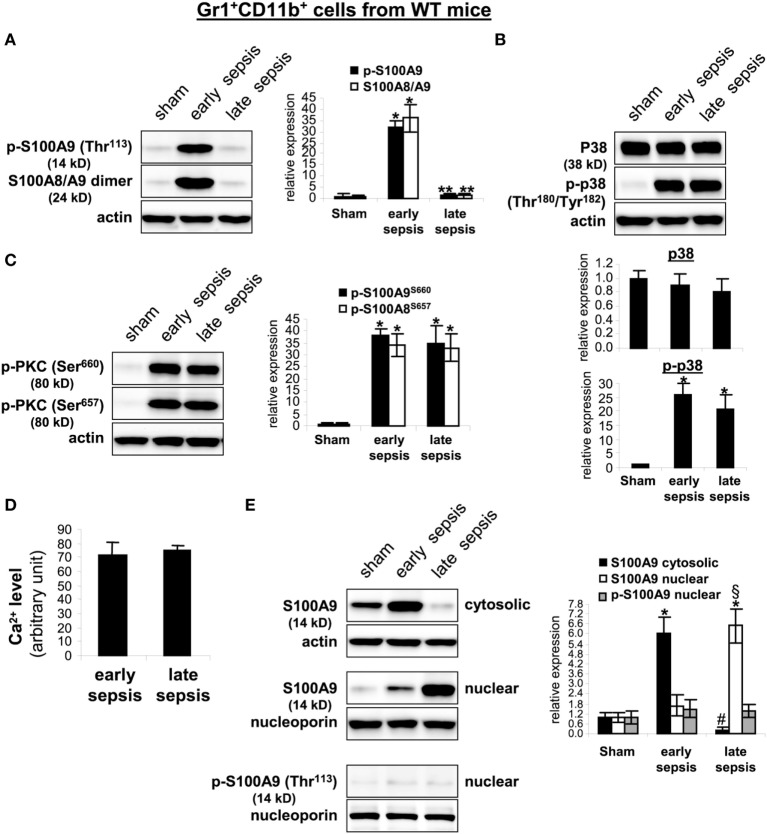
The S100A9 protein phosphorylation and dimerization with S100A8 is inhibited and translocated to the nucleus in Gr1^+^CD11b^+^ cells during late sepsis. Gr1^+^CD11b^+^ cells were isolated from the bone marrow cells by positive selection. The early and late sepsis groups, respectively, included mice that were killed between days 1–5 and 6–28 after cecal ligation and puncture. Cell lysates were prepared, and expression of the indicated proteins was determined by immunoblotting. **(A)** S100A9 phosphorylation was probed with anti-phospho threonine 113 antibody. To detect the S100A8/A9 heterodimer, the lysate was resolved under non-denaturing conditions which produced a protein band of ~24 kDa. **(B)** Levels of p38 MAPK phosphorylation were determined using anti-phospho tyrosine antibody that recognizes tyrosine number 180 and 182. **(C)** Levels of phosphorylated protein kinase C were determined using anti-phospho serines 643 and 660 antibodies. **(D)** Intracellular calcium levels were measured using a cell-based Fluo-4 NW calcium assay. Data are means ± SD of four mice per group and are representative of two experiments. **(E)** Localization of the S100A9 protein. Nuclear and cytosolic proteins were extracted from Gr1^+^CD11b^+^ cells and immunoblotted with the S100A9 antibody. Nuclear extracts were probed for p-S100A9. Membranes were re-probed with actin or nucleoporin antibody as a loading control. In **(A–C,E)**, cells were pooled from 2–3 mice per group. Representative blots [left; **(A,C,E)**; upper, **(B)**] and densitometric analysis of blots from three experiments are shown. Values were normalized to β-actin or nucleoporin, and are presented relative to sham [*p < 0.001 vs. sham; **p < 0.001 vs. early sepsis; ^#^*p* < 0.001 vs. early sepsis S100A9 cytosolic; ^§^*p* < 0.02 vs. early sepsis S100A9 nuclear **(E)**].

To probe what prevents S100A8/A9 release from late sepsis Gr1^+^CD11b^+^ cells, we examined S100A9 protein phosphorylation and subcellular localization. S100A9 protein is phosphorylated by p38 MAPK (32, 33) on threonine113 at the C-terminal domain in a Ca^2+^-dependent manner (34), a modification that facilitates translocating S100A8/A9 complex from the cytosol to plasma membrane for secretion (35). Immunoblotting showed that S100A9 protein was phosphorylated on threonine 113 and formed heterodimers with S100A8 in early, but not late sepsis Gr1^+^CD11b^+^ cells (Figure 7A), despite activation of p38 MAPK (Figure 7B).

Secretion of S100A8/A9 complexes is also an energy-dependent process, requires activation of PKC and Ca^2+^-dependent signaling to support S100A8/A9 interactions with microtubules and translocation to plasma membrane and subsequent release (16). We detected similar phosphorylation (activation) of PKC in early and late sepsis Gr1^+^CD11b^+^ cells (Figure 7C). In addition, Ca^2+^ binding increases the stability of the S100A8/A9 protein complexes (36). An assay of intracellular calcium did not reveal significant differences in the Ca^2+^ levels between early and late sepsis Gr1^+^CD11b^+^ cells (Figure 7D). We also examined subcellular localization of S100A8/A9 proteins. S100A8/A9 proteins are mainly localized in the cytosol (35). As shown in Figure 7E, S100A9 protein was mainly detected in the cytosol in Gr1^+^CD11b^+^ cells in early sepsis, but was mainly localized in the nucleus in late sepsis. We did not detect phospho-S100A9 protein in the nucleus by western blot in early or late sepsis Gr1^+^CD11b^+^ cells (Figure 7E), suggesting that S100A9 protein accumulates in the nucleus in an unphosphorylated form. In addition, we detected S100A8 protein mainly in the cytosol in early and late sepsis Gr1^+^CD11b^+^ cells, but its levels were markedly reduced in late sepsis cells (data not shown), likely due to lack of S100A9 in the cytosol. Together, these results suggest that nuclear translocation of S100A9 in Gr1^+^CD11b^+^ MDSCs during late sepsis prevents its secretion and pro-inflammatory effects.

### MDSCs Lacking S100A9 Do Not Express miR-21 and miR-181b during Late Sepsis

**Figure 8:**
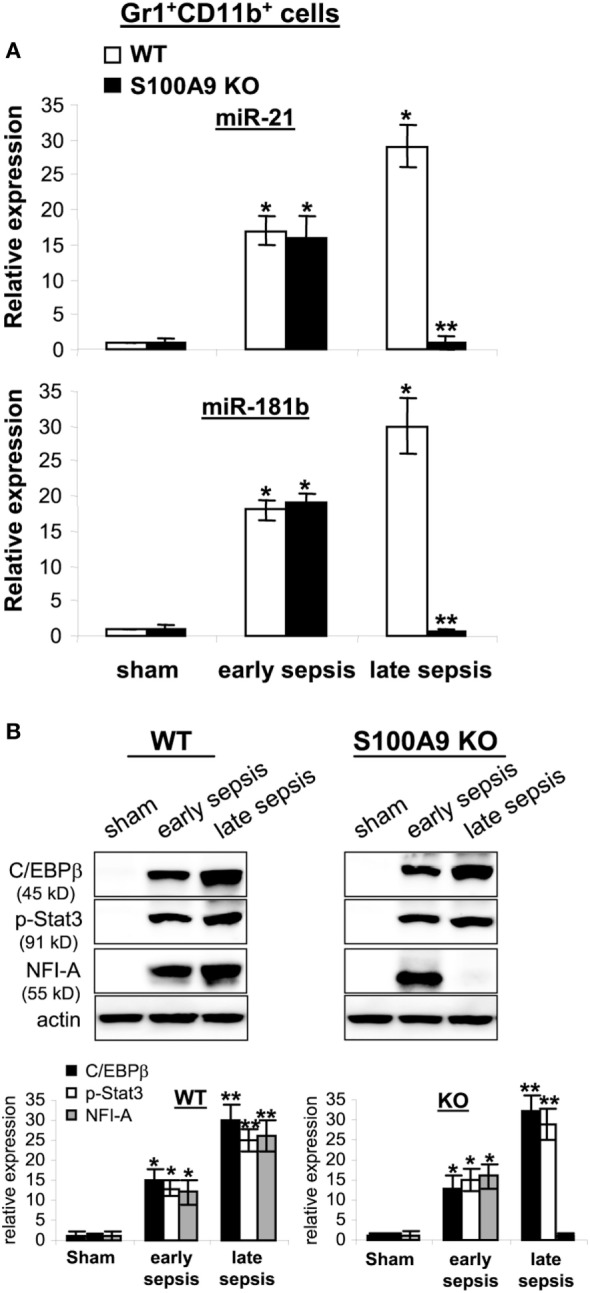
The expression of miR-21 and miR-181b in Gr1^+^CD11b^+^ cells is inhibited during late sepsis. The Gr1^+^CD11b^+^ cells were isolated from the bone marrow of sham and septic mice using magnetic beads. The early and late sepsis groups, respectively, included mice that were killed between days 1–5 and 6–28 after cecal ligation and puncture. **(A)** Measurements of miR-21 and miR-181b expression. MiRNA-enriched RNA was isolated, and levels of miR-21 and miR-181b were determined by quantitative real-time qPCR using miR-21 and miR-181b specific assay primers. Sample values were normalized to U6 RNA as an internal control. Data are means ± SD (*n* = 3–5 mice per group), presented relative to values from sham mice (set at onefold). Results are representative of three experiments (*p < 0.001 vs. sham; **p < 0.001 vs. late sepsis WT). **(B)** Protein levels C/EBPβ, p-Stat3 and NFI-A. Gr1^+^CD11b^+^ cell lysates were prepared and immunoblotted with antibodies against the indicated proteins. Representative blots (upper) and densitometric analysis of blots (lower) from three experiments is shown. Values were normalized to β-actin, and are presented relative to sham (*p < 0.01 vs. sham; **p < 0.05 vs. early sepsis).

Expression of miR-21 and miR-181b is induced in Gr1^+^CD11b^+^ cells during sepsis and promotes Gr1^+^CD11b^+^ cell expansion (24). We reported that blocking miR-21 and miR-181b in septic mice by administration of miRNA antagomiRs diminishes Gr1^+^CD11b^+^ MDSC expansion during late sepsis response (24). Accordingly, we measured miR-21 and miR-181b levels by RT-PCR in early and late sepsis. Levels of miR-21 and miR-181b in Gr1^+^CD11b^+^ cells were increased during early sepsis in both wild-type and knockout mice (Figure 8A). In late sepsis Gr1^+^CD11b^+^ cells, both miRNAs further increased in wild-type mice, but diminished in S100A9 knockout mice.

We previously reported that miR-21 and miR-181b induction during sepsis is dependent on both C/EBPβ expression and Stat3 phosphorylation, which synergize to activate miR-21 and miR-181b promoters (37). To determine whether decreased miR-21 and miR-181b expression in S100A9 knockout mice during late sepsis is due to lack of C/EBPβ expression and/or Stat3 phosphorylation, we examined C/EBPβ and phosphorylated Stat3 protein levels in the Gr1^+^CD11b^+^ cell lysates. C/EBPβ expression and Stat3 phosphorylation were similarly induced in wild-type and S100A9 knockout mice (Figure 8B). We also reported that NFI-A expression is induced downstream of miR-21 and miR-181b and promotes Gr1^+^CD11b^+^ cell expansion during sepsis by attenuating myeloid cell differentiation and maturation (31). Here, we detected NFI-A in Gr1^+^CD11b^+^ cells from early, but not late septic S100A9 knockout mice (Figure 8B). These results strongly support that S100A9 sustains both NFI-A and miR-21 and miR-181b levels during late sepsis immunosuppression.

### Administration of S100A8/A9 to S100A9 Knockout Mice Has No Impact on Sepsis Response

**Figure 9:**
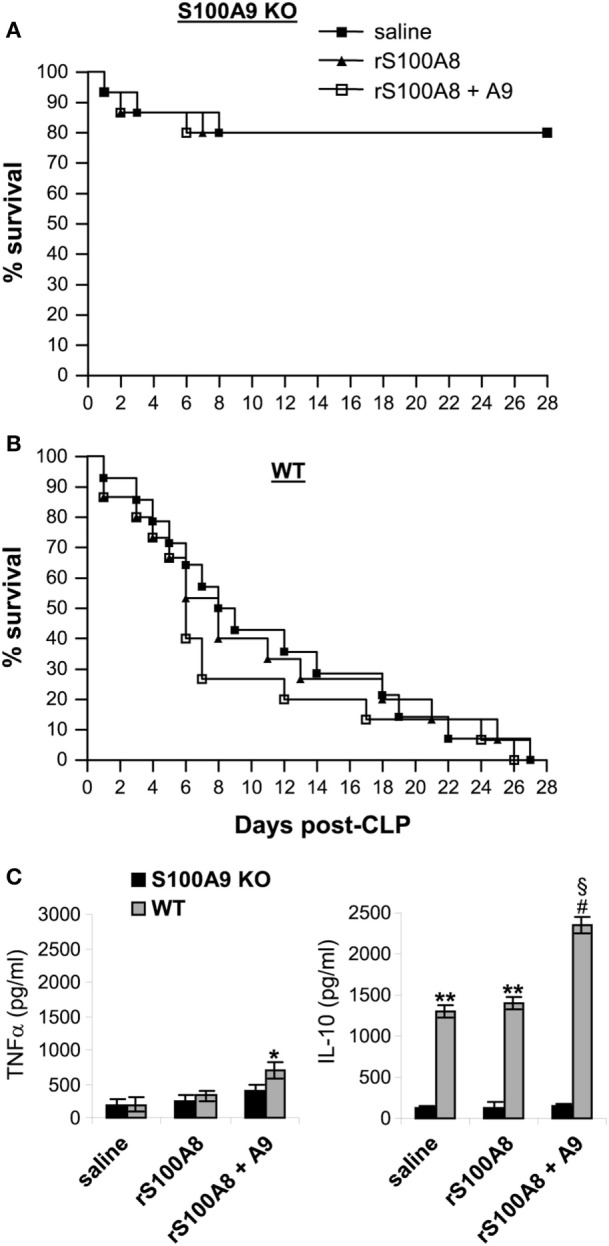
Administration of rS100A8/9 into the S100A9 knockout mice undergoing sepsis has no effect on sepsis survival or inflammatory cytokine production. Sepsis was induced by cecal ligation and puncture (CLP), and mice were treated as described in Figure 2. On day 5 and day 6 after CLP, mice were injected (i.p.) with 50 µg each of recombinant mouse S100A8 and/or S100A9 protein or 100 µl saline. **(A,B)** Kaplan–Meier survival curves showing deaths during the early and late sepsis phases in KO and WT mice (*n* = 15 per group). **(C)** Plasma levels of TNFα and IL-10. Blood was collected at day 15 after CLP from the KO mice (*n* = 6 mice per group) and at days 7–18 from the WT mice (*n* = 4–6 mice per group). Plasma was collected, and levels of TNFα and IL-10 were determined by ELISA. Data are means ± SD. *p < 0.05 vs. KO; **p < 0.001 vs. KO; ^#^*p* < 0.0001 vs. KO; ^§^*p* < 0.01 vs. saline or rS100A8 WT. r, recombinant.

S100A9 integrates two signaling pathways that facilitate S100A8/9 release and may functionally dominate complex function (35). Other studies suggested that S100A8 may be dominant in the S100A8/A9 complex (21). Because S100A8 protein is diminished in the S100A9 knockout mice (Figure 2C), we examined whether reconstitution of the S100A9 knockout mice with S100A8 and/or S100A9 affects late sepsis responses. We injected (i.p.) the wild-type and S100A9 knockout mice with recombinant mouse S100A8 and/or S100A9 at day 5 after CLP (i.e., at the end of early sepsis phase). Administration of S100A8 alone or in combination with S100A9 did not affect survival of the S100A9 knockout mice (Figure 9A). In the wild-type mice, only injection of S100A8/A9 increased mortality by ~24–16% between days 6 and 11 compared with mice injected with saline (Figure 9B). In addition, we did not detect significant changes in plasma levels of immunosuppressive cytokine IL-10 in mice treated with S100A8 or S100A8/A9, but levels of pro-inflammatory TNFα slightly increased in mice treated with S100A8/A9 compared with S100A8 alone (Figure 9C). In the wild-type mice, S100A8/A9 injection slightly, but significantly, increased TNFα production. Of note, levels of IL-10 were significantly higher compared with the S100A9 knockout mice, and were further elevated after S100A8/A9 injection. These results suggest that S100A8 absence in the S100A9 knockout mice does not affect the inflammatory response to sepsis. These results also suggest that administration of S100A8/A9 into wild-type mice undergoing late sepsis can further enhance immunosuppression.

### S100A8/A9 Plasma Levels Decrease in Chronic Septic Patients, but Remain Elevated within Phagocytes

**Figure 10:**
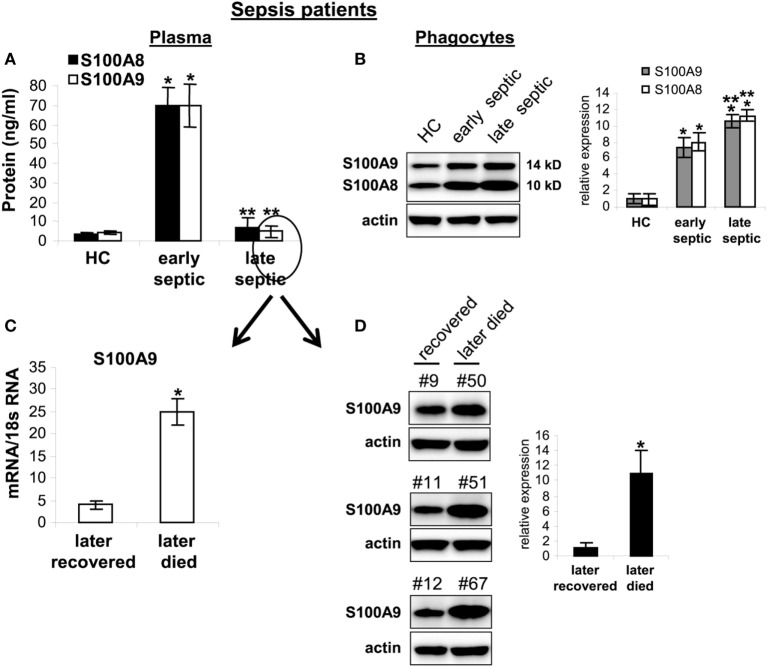
Release of S100A8/A9 proteins is blocked in late septic patients. Blood phagocytes and plasma were isolated by gradients of Histopage-1077 and Histopaque-1119 (Sigma). **(A)** Levels of S100A8/A9 proteins in plasma were determined by ELISA (run in duplicate). Data are means ± SD (*n* = 8 subjects/group) (*p < 0.001 vs. HC; **p < 0.001 vs. early septic). **(B)** levels of S100A8/A9 proteins in phagocytes lysates were determined by western blot. Representative blot (left) and densitometric analysis of blots (right) are shown. Results are representative of three immunoblots (*p < 0.01 vs. HC; *p < 0.05 vs. early septic). **(C,D)** The late septic patient group shown in panel **(A)** was divided into two sub-groups: those who later recovered (*n* = 5) from sepsis or died (*n* = 3). Their frozen phagocytes were divided. A portion of the cells was used for RNA extraction and determination of S100A8/A9 mRNA levels by real-time PCR, and expression levels were normalized to 18S rRNA **(C)** (*p < 0.001). The remainder of the cells were lysed and S100A9 protein levels were determined by western blot **(D)**. Representative blots (left) and densitometric analysis of blots (right) are shown. Results are representative of three immunoblots (*p < 0.01). Patient numbers are shown on top of the blots. Early septic included patients who were within 1–5 days after diagnosis. Late septic included patients who have been septic for 6–31 days. HC, health control.

Secreted S100A8/A9 may promote acute and/or chronic inflammation (18). To determine whether S100A8/A9 expression in sepsis patients correlates with sepsis inflammation, we first measured plasma S100A8/A9 during human sepsis. Patients were divided into two groups: the early septic group included patients within 1–5 days of clinically detected sepsis and the chronic sepsis group had been septic at least 6 days and up to 31 days. Figure 10A shows significant increases in S100A8/A9 plasma levels in the early septic group compared with healthy controls. Notably, circulating S100A8/A9 levels decreased in late septic patients. We then determined whether the decrease in plasma levels of S100A8/A9 proteins correlated with reduced protein expression by phagocytes. Using western blotting, we observed marked increases in S100A8/A9 proteins in blood phagocytes from early and late septic patients compared with healthy controls (Figure 10B). We further examined whether S100A9 expression correlates with sepsis prognosis for the late septic group. Both mRNA and protein levels of S100A9 markedly decreased in phagocytes from patients who later recovered from chronic sepsis but remained elevated in those who eventually died (Figures 10C,D). Together, these results suggest that sustained intracellular S100A9 in phagocytes and perhaps MDSCs promote chronic sepsis by sustaining immunosuppression.

## Discussion

It is increasingly evident that MDSCs promote sepsis-induced immunosuppression in mice (30, 38) and their counterparts may contribute to the profound and sustained immunosuppression in human sepsis (7). Despite decades of studies on sepsis pathobiology and multiple therapeutic failures, sepsis is still a health-care crisis without available targeted treatment options (8, 39). Importantly, the mediators that sustain MDSC generation and immunosuppression in chronic sepsis remain a mystery. This study, for the first time, introduces the concept that S100A9 protein contributes to late sepsis mortality in mice by its sustained expression and nuclear retention. Tantamount to molecular targeting therapeutics, genetic deletion of S100A9 in mice improves chronic sepsis mortality. That this new paradigm may translate to human sepsis, we show that S100A8/A9 intracellular mRNA and protein levels in phagocytes are elevated in patients with more chronic sepsis, and that a correlation may exist between this repressor like phenotype and human sepsis mortality. However, more research in humans is needed to test this new theory before targeted therapy is developed. The emerging use of immune checkpoint, as well as metabolic checkpoint, drugs (40, 41) may allow translation of our concept into both a better understanding and treatment of human sepsis—a major gap and health-care need.

We detected significantly elevated levels of S100A8/A9 proteins in the plasma of early septic mice, a known feature of S100 proteins as pro-inflammatory, acute phase proteins (15, 16). However, we showed that S100A8/A9 proteins in septic mice decreased in the late sepsis phase, despite elevated levels of mRNAs and proteins in circulating phagocytes and Gr1^+^CD11b^+^ MDSCs in the bone marrow and spleens. Another important observation supporting our paradigm was decreases in immunosuppressive IL-10 cytokine and increased microbial clearance from peritoneum in S100A9 knockout mice during late sepsis. This supports that S100A9 directs immune suppression in phagocytes and MDSCs. However, the mechanistic link between miR-21 and miR-181b expression, as well as the signaling regulatory path involving C/EBPβ and NFI-A, supports that S100A9 acts as a transcription co-factor or an indirect epigenetic mediator. Epigenetic pathways like those regulated by NAD^+^ Sirtuins 1 and 6 (12) are candidates, as well as direct cooperativity with transcription repressor complexes like RelB (42, 43).

Our study does not refute the substantive reports showing S100A8/A9 proteins function mainly as extracellular pro-inflammatory mediators (14–16). A recent study showed that S100A8/A9 proteins promote septic shock in mice *via* activating TLR4 on innate immunity cells (21). Other findings support that S100A8/A9 proteins also exert anti-inflammatory effects (22), and our data suggest a dual role for S100A9 protein. Intracellular S100A9 may act as an anti-inflammatory/immunosuppressive mediator through reprogramming the Gr1^+^CD11b^+^ myeloid cells into MDSCs, whereas extracellular S100A9 may promote inflammation in the plasma and when secreted by phagocytes. This interpretation of dual functioning proteins is not without precedent. For example, we previously discovered that HMGB1 histone binding protein, which like S100A9 induces inflammation after release and through stimulation of TLR4, is also an epigenetic repressor during endotoxin tolerance in human monocytes, a biomarker of immunosuppression (44). Of note, administration of recombinant S100A8 and/or S100A9 at the onset of the late sepsis phase did not impact sepsis outcomes in the S100A9 knockout mice. However, S100A8/A9 enhanced mortality in the wild-type mice for the first few days after the injection, and this response was accompanied by increases in the levels of immunosuppressive IL-10 production.

Many MDSCs accumulate during late sepsis in mice (30, 38). Our finding that S100A9 knockout mice did not generate immunosuppressive Gr1^+^CD11b^+^ cells (i.e., MDSCs) during late sepsis response is physiologically significant, and supports that S100A9 is necessary for chronic MDSC repressor function. Moreover, S100A9 knockout mice in early sepsis response were still able to generate normal (immune competent) Gr1^+^CD11b^+^ cells, similar to wild-type mice. While these functionally competent myeloid cells were phenotypically similar to the MDSCs generated in late sepsis, they did not suppress T cell activation or proliferation. Thus, S100A9 protein reprograms immature Gr1^+^CD11b^+^ myeloid cells into MDSCs in late sepsis, but has no impact on myeloid cell phenotype or functions under normal conditions or during the early phase of sepsis.

S100A9 protein phosphorylation and dimerization with S100A8 and subsequent secretion require phosphorylation on S100A9 by p38 MAPK (32, 33) and activation of PKC in a Ca^2+^-dependent manner (16). Our results showed that S100A9 protein phosphorylation and dimerization were inhibited in the Gr1^+^CD11b^+^ cells from late, but not early, septic mice despite normal activation of p38 and PKC throughout the sepsis course. In addition, we did not observe changes in the intracellular Ca^+2^ levels. Most importantly, we found that S100A9 protein was mainly localized in the nuclear compartment in late sepsis Gr1^+^CD11b^+^ cells (i.e., MDSCs). S100A8/A9 proteins are known to have diverse functional properties based on location and posttranslational phosphorylation/dephosphorization mechanisms (16). It is unclear from the current study how S100A9 protein is translocated into the nucleus in late sepsis cells, since a nuclear localization signal has not been reported, to the best of our knowledge. Since S100A9 phosphorylation promotes its translocation from the cytosol to the plasma membrane for secretion and increases its Ca^2+^ binding property (16, 45), it is possible that nuclear translocation only occurs in the dephosphorylated state in which calcium is unbound. Testing that possible mechanism will require genetic and therapeutic targeting.

In summary, this study for the first time identifies a novel chronic immune repressor mechanism in MDSCs, which may have untoward consequences on sepsis resolution during the PICS syndrome (46). Unanswered important questions include what controls S100A9 translocation to the nucleus, what disrupts its secretion? and whether this novel path can be a therapeutic target?

## Ethics Statement

All animal experiments were conducted in accordance with National Institutes of Health guidelines and were approved by the East Tennessee State University Animal Care and Use Committee (Protocol #: 160704). The human study was approved by the Institutional Review Board (IRB) of the East Tennessee State University (IRB#: 0714.6s). Signed informed consent was obtained from all subjects.

## Author Contributions

JD and AK conducted and analyzed the experiments; DY recruited patients and collected blood samples; CM edited the manuscript; ME designed the project, supervised research, and wrote the manuscript.

## Conflict of Interest Statement

The authors declare that the research was conducted in the absence of any commercial or financial relationships that could be construed as a potential conflict of interest.

## Footnotes

^1^http://www.komp.org.

^2^http://www.mousebiology.org.
