# Supplementary material for: Intracellular S100A9 Promotes Myeloid-Derived Suppressor Cells during Late Sepsis
Source: Front Immunol. 2017 Nov 17;8:1565. doi: 10.3389/fimmu.2017.01565 (PMC5698275; doi:10.3389/fimmu.2017.01565)
Supplement: Supplementary file 1 [file Table_1.PDF]

Table S1. CLP mice that were used in this study.

Time after CLP	Wild-type		S100A9 KO	
(days)	early sepsis	late sepsis	early sepsis	late sepsis
1	1		3	
2	4		2	
3	6		2	
4	5		5	
5	3		1	
6		3		3
7		3		3
8		1		1
9		2		2
10		3		3
11		1		1
12		3		3
13		2		2
14		2		2
15		3		3
16		2		2
17		1		1
18		3		3
19		2		2
20		3		3
21		1		1
22		0		0
23		2		2
24		2		2
25		3		3
26		2		2
27		1		1
28		0		0
total	19	45	13	45

Note, for each moribund (dying) mouse from the wild-type group, a healthy appearing mouse from the knockout group was also sacrificed and analyzed, but is reported as “survivor.” Mice that spontaneously died (found dead) are not included.
